# Role of the mTOR pathway in minor salivary gland changes in Sjogren’s syndrome and systemic sclerosis

**DOI:** 10.1186/s13075-018-1662-4

**Published:** 2018-08-04

**Authors:** Zeki Soypaçacı, Zeynep Zehra Gümüş, Fulya Çakaloğlu, Mustafa Özmen, Dilek Solmaz, Sercan Gücenmez, Önay Gercik, Servet Akar

**Affiliations:** 1 Department of Internal Medicine, Division of Nephrology, Izmir Katip Celebi University School of Medicine, Karabağlar, 35360 İzmir, Turkey; 2 Dogubeyazit Public Hospital, Internal Medicine, Agri, Turkey; 3 Department of Pathology, Izmir Katip Celebi University, Izmir, Turkey; 4 Department of Internal Medicine, Division of Rheumatology, Izmir Katip Celebi University, Izmir, Turkey

**Keywords:** Target of rapamycin proteins, mTOR pathway, Sjogren’s syndrome, Systemic sclerosis, PTEN protein, Human, Transforming growth factor beta

## Abstract

**Background:**

To examine the activity of the mammalian target of rapamycin (mTOR) pathway and its regulators, transforming growth factor (TGF)-β1 and phosphatase and tensin homolog (PTEN), in minor salivary gland biopsies of Sjogren’s syndrome (SS) and systemic sclerosis (SSc) patients.

**Methods:**

We retrospectively evaluated SS, SSc, and SS-SSc overlap patients admitted to our outpatient rheumatology clinic between January 2007 and December 2015 who underwent a minor salivary gland biopsy. Patient demographics and some clinical features were obtained from hospital records. Immunohistochemistry was used to analyze total mTOR, total PTEN, and TGF-β1 expression in the biopsied tissues. The biopsy specimens were also examined for the presence and degree of fibrosis.

**Results:**

Minor salivary gland biopsies of 58 SS, 14 SSc, and 23 SS-SSc overlap patients were included in the study. There was no significant difference in mTOR expression between these groups (*P* = 0.622). PTEN protein was expressed in 87.2% of patients with SS, 57.9% with overlap syndrome, and 100% of the SSC patients, and these differences were statistically different (*P* = 0.023). Although ductal epithelial TGF-β1 expression was similar between the groups (*P* = 0.345), acinar cell expression was found to be more frequent in the SSc (72.7%) and overlap patients (85.7%) in comparison with the SS cases (58.2%; *P* = 0.004).

**Conclusion:**

mTOR may be one of the common pathways in the pathology of both SS and SSc. Hence, there may be a role for mTOR inhibitors in the treatment of both diseases. Additionally, PTEN and TGF-β1 expression may be a distinctive feature of SSc.

## Background

Sjogren’s syndrome (SS) is a chronic, systemic, inflammatory disease [1]. The characteristic pathologic findings for this disorder are lymphocytic infiltration of the exocrine glands leading to autoantibody production and tissue destruction [1, 2]. Consistent with its pathogenesis, the first symptoms of SS are generally xerostomia and keratoconjunctivitis sicca [2]. SS can occur as an isolated primary condition or secondary to another connective tissue disease. At the beginning of SS onset, CD4-positive T helper cells play a pathogenic role whereas, in late-term SS, B cells play a predominant role. Recent studies have indicated that epithelial cells are central to autoimmune pathways where they produce human leukocyte antigen (HLA), adhesion and costimulatory molecules, and cytokines and chemokines [2]. The term ‘autoimmune epitheliitis’ has thus been suggested to describe the etiology of SS.

Systemic sclerosis (SSc) is a chronic autoimmune disease characterized by increased fibrosis and slightly enlightened pathogenesis [3, 4]. The most frequent skin sign of SSc is dermal infiltration by myofibroblasts that synthesize type I collagen and alpha smooth muscle actin (α-SMA) [4]. Increased profibrotic mediators, such as transforming growth factor (TGF)-β, and increased mammalian target of rapamycin (mTOR) activity have also been reported in dermal fibroblasts of SSc patients [4, 5]. mTOR is a serine kinase that plays a role in the regulation of cell growth and proliferation. The mTOR complex includes two multiprotein complexes, mTOR complex 1 (mTORC1) and mTOR complex 2 (mTORC2) [6]. mTORC1 activates S6 kinase 1 (S6K1) and eukaryotic translation initiation factor 4E (eIF4E) which are responsible for mRNA translation [5]. mTOR also regulates cell survival and is stimulated by growth factors, nutrients, stress signals, phosphoinositol-3-kinase (PI3K), mitogen-activated protein kinase (MAPK), adenosine monophosphate (AMP), and adenosine monophosphate-activated protein kinase (AMPK). mTORC2 regulates the actin cytoskeleton and activates protein kinase C (PKC)-α and Akt (protein kinase B, or PKB) [6]. mTOR multiprotein complexes have a positive effect on fibrotic interleukins (ILs). Liang et al. reported previously that IL-4, IL-6, IL-17, and TGF-β are downregulated after mTOR inhibition with rapamycin [5]. Phosphatase and tensin homolog (PTEN) is also involved in the regulation of mTOR activity and usually inhibits mTOR via the inhibition of Akt [7]. Decreased intracellular levels of PTEN cause PI3K/Akt/mTOR pathway activation and increase cell proliferation, survival, adhesion, migration, and angiogenesis [7]. Another mTOR regulator molecule, TGF-β, activates intracellular signaling pathways such as PI3K/Akt/mTOR and SMAD [8, 9]. TGF-β can also both enhance and suppress PTEN, an effect that depends on Ras/ERK pathway activation [7].

Multiple signaling pathways such as MAPK, Akt, NF-κB, Bcl-2, and JAK/STAT are found to be activated in systemic diseases in which the mTOR pathway is also an attractive therapeutic target [10]. The relationship between mTOR and increased skin fibrosis in SSc has previously been investigated both in vivo and in vitro. The role of rapamycin, an mTOR inhibitor, was investigated previously in only a murine model of SS, the results of which suggested that it has therapeutic potential [11]. We therefore aimed, in our current study, to investigate the role of the mTOR pathway in the pathologic changes observed in minor salivary gland biopsies (MSGBs) from SS, SSc, and SS/SSc overlap syndrome patients.

## Methods

### Patients and data collection

Patients admitted to the outpatient rheumatology clinic in our tertiary hospital between January 2007 and December 2015 were retrospectively reviewed. These patients were divided into SSc, SS, and SSc/SS overlap subgroups. Demographic (age, gender), clinical (duration of disease, presence of sicca symptoms, Schirmer’s and tear breakup time (BUT) test results), and serum autoantibody data for these cases were collected using their medical records. Patients who answered positively to at least one of the questions regarding keratoconjunctivitis sicca and xerostomia were considered positive for sicca symptoms. A BUT test result < 10 s and a Schirmer’s test finding ≤ 5 mm/5 min were also considered positive indicators of sicca symptoms.

SS and SSc patients aged ≥ 18 years who fulfilled the American-European Consensus Group (AECG) classification criteria for Sjogren’s syndrome [12] and the ACR/EULAR 2013 criteria for systemic sclerosis [13], respectively, and who underwent MSGB were screened for inclusion in the study cohort. All of the SSc patients who underwent MSGB had at least one sicca symptom or had a positive autoantibody related to sicca symptoms. We excluded patients who had received any previous treatment with mTOR inhibitors. We also excluded any secondary SS or SSc patients other than overlap cases. We did not use an informed consent form since this was a retrospective study.

### Histopathological evaluation

The MSGBs were fixed with 10% buffered formalin for at least 6 h and then monitored with a closed-loop tissue monitoring machine overnight. Serial sections of 4–5 μm thickness were obtained from a single paraffin-embedded block for each patient, stained with hematoxylin and eosin (H&E), and examined under a light microscope by a single expert pathologist (FC). The specimens were evaluated for the presence and number of lymphocytic foci, the presence and grade of fibrosis, and lobular or acinar atrophy. Focal lymphocytic sialadenitis with a score of ≥ 1 foci/4 mm^2^ was accepted as diagnostic for SS [14]. The extent of fibrosis in the MSGB specimens was assessed semiquantitatively and graded as mild (less than 25% of the surface area), moderate (between 25 and 50%), or severe (> 50%).

### Immunohistochemical staining

The MSGB specimens were evaluated by immunohistochemical (IHC) staining with antibodies for total PTEN (Spring Bioscience Rabbit Anti-Human PTEN Rabbit Monoclonal, Clone SP170), total mTOR (Spring Bioscience Rabbit Anti-Human mTOR Polyclonal Antibody), and TGF-β1 (Spring Bioscience Rabbit Anti-Human Transforming Growth Factor 1β Polyclonal Antibody) by a single expert pathologist (FC). IHC staining slides were evaluated via light microscopy. The positive controls used in this study were prostate adenocarcinoma tissue for PTEN, placenta for TGF-β1, and breast cancer tissue for mTOR.

### Immunohistochemical evaluation

**Fig. 1:**
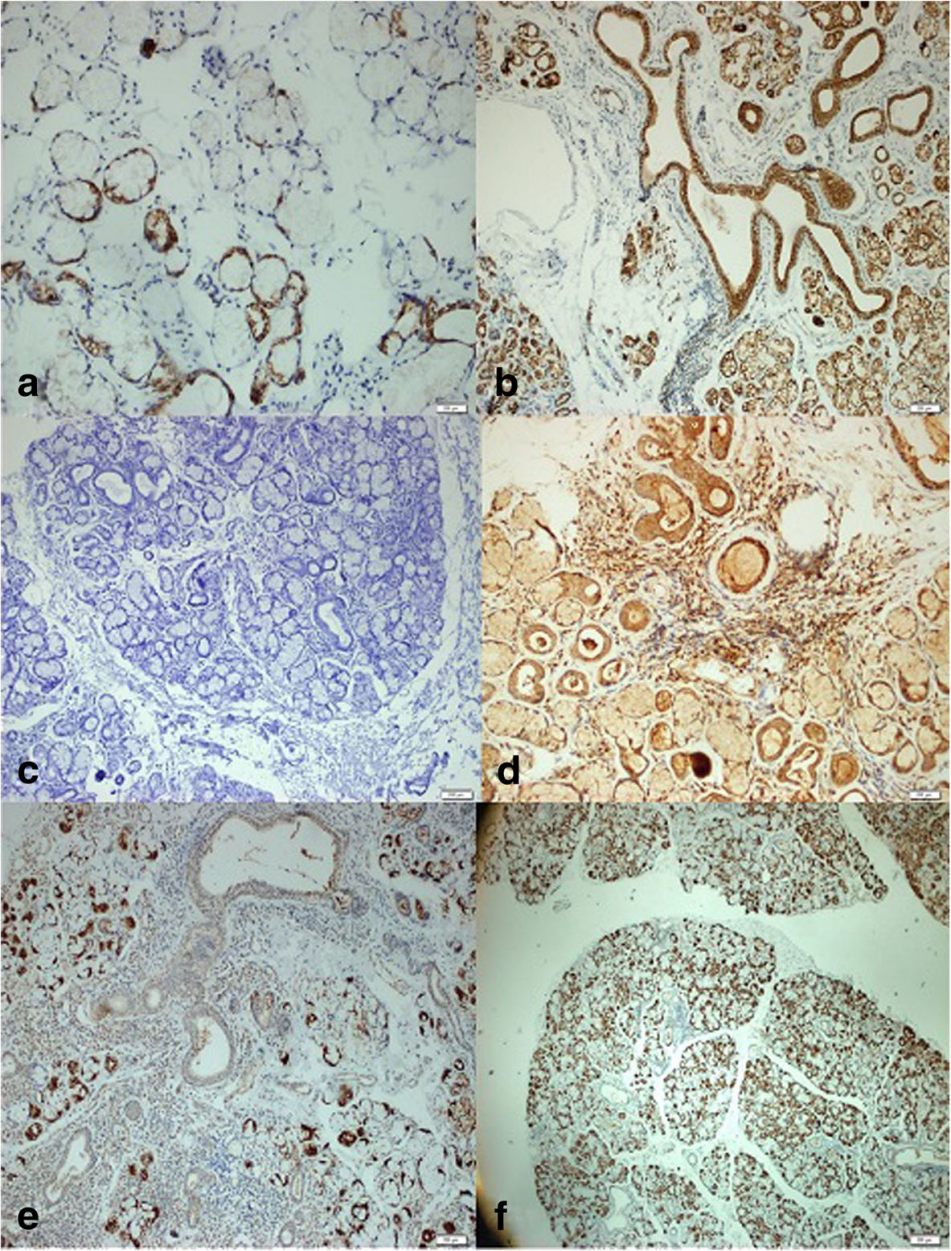
Immunohistochemical staining of mTOR, PTEN, and TGF-β1 in minor salivary gland biopsies. Representative samples showing mild (**a**) and strong (**b**) positivity for mTOR, negative (**c**) and strong positivity for PTEN (**d**), and mild (**e**) and strong positivity for TGF-β staining (**f**). Positive staining for TGF-β was mainly observed in the acinar regions of the salivary glands

IHC staining of mTOR was semiquantitatively assessed as mild (1+) (Fig. 1a), moderate (2+), or strong (3+) (Fig. 1b) positivity [15]. IHC staining of PTEN was graded as negative (Fig. 1c), or as mild (1+) or strong (2+) positivity (Fig. 1d) [16]. IHC staining of TGF-β1 was semiquantitatively assessed and graded between 0 and 4 according to the level of staining [17] (Fig. 1e, f).

### Statistical analysis

Unless otherwise stated, results are presented as a mean and standard deviation (SD) or percentage as appropriate. Comparisons of categorical data between groups were made using the chi-square test. The Spearman’s rank correlation test was performed for bivariate correlations between variables. All tests were two-tailed and a *P* value of < 0.05 was considered statistically significant for all measurements. All statistical analyses were made using the Statistical Package of Social Science (SPSS) version 16.0 software (Chicago, IL).

## Results

### Patient demographic and baseline features

Demographic and baseline features of the patients are presented in Table 1. Formalin-fixed MSGB sections from 58 patients with SS, 16 with SSc, and 23 with SSc/SS overlap syndrome were initially included in the study samples. However, two female SSc samples had to be excluded to due to difficulties with IHC staining. As expected, the frequency of sicca symptoms was higher in the SS patients (90%) than in the SSc (77%) or overlap syndrome cases (70%). BUT test positivity was determined as 60% in the SS patients, 36% in the SSc patients, and 74% in the overlap syndrome patients. Schirmer test positivity was found to be 58% in the SS patients, 50% in the SSc patients, and 58% in the overlap syndrome patients. Anti-Ro (SSA) or anti-La (SSB) positivity was higher in the SS patients (96%) than SSc (55%) and overlap syndrome (71%) patients. Examination of the biopsy specimens revealed a focus score of ≥ 1 foci/4 mm^2^ in 52 out of the 58 (90%) SS patients, 22 out of the 23 (96%) overlap patients, and none of the SSc patients.

**Table 1:** Demographic and baseline features of the patients

	Sjogren’s syndrome (*n* = 58)	Systemic sclerosis (*n* = 14)	Overlap syndrome (*n* = 23)
Age, years (mean ± SD)	52.6 ± 13.1	53.9 ± 14.2	48.0 ± 9.3
Duration of disease, months (mean ± SD)	58.3 ± 23.4	43.0 ± 23.9	56.8 ± 35.4
Sicca symptom positivity, %	90	77	70
Schirmer test positivity, %	58	50	58
Breakup time test positivity, %	60	36	74
Anti-Ro (SSA) or La (SSB) positivity, %	96	55	71

### mTOR, PTEN, and TGF-β1 expressions

mTOR expression was evident in 94% of the SS group, 100% of the overlap cases, and 91% of the SSc patients (Table 2). There were no significant differences in the presence (*P* = 0.462) or degree (*P* = 0.622) of mTOR expression between these groups. PTEN protein expression was detected in 87% of the SS patients, 58% of the overlap cases, and 100% of the SSc patients (Table 2) with significant differences in the presence (*P* = 0.004) and intensity (*P* = 0.023) of staining. Although the ductal epithelial TGF-β1 expression was similar between the groups (*P* = 0.345), acinar cell expression (Table 2) was more frequent in the SSc (73%) and overlap patients (86%) in comparison with the SS (58%) cases with borderline significance (*P* = 0.05). Additionally, more of the acinar TGF-β1 staining was strongly positive in SSC patients (46% vs 19% and 4%; *P* = 0.004).

**Table 2:** Immunohistochemical staining results in the three patient groups (mTOR, PTEN, and TGF-β1 in acinus)

		Negative	Mild positivity	Moderate positivity	Strong positivity
mTOR	SS	3 (6)	25 (46)	20 (37)	6 (11)
	Overlap syndrome	0	11 (55)	9 (45)	0
	SSc	1 (9)	4 (36)	5 (46)	1 (9)
PTEN	SS	6 (13)	23 (49)	–	18 (38)
	Overlap syndrome	8 (42)	5 (26)	–	6 (32)
	SSc	0	7 (58)	–	5 (42)
TGF-β1	SS	23 (42)	20 (36)	10 (18)	2 (4)
	Overlap syndrome	3 (14)	10 (48)	4 (19)	4 (19)
	SSc	3 (27)	2 (18)	1 (9)	5 (46)

All values are shown as *n* (%)

*mTOR* mammalian target of rapamycin, *PTEN* phosphatase and tensin homolog, *SS* Sjogren’s syndrome, *SSc* systemic sclerosis, *TGF* transforming growth factor

### Fibrosis features

In general, fibrosis was evident in all of our patient groups but we did not observe severe fibrosis in any MSGB sections. There was also no significant difference found between the study groups in terms of the presence of fibrosis (*P* = 0.833). Correlation analysis between the immunohistochemical staining results, presence of fibrosis, and the demographic, clinical characteristics, and autoantibodies of the SS patients showed only a negative correlation between PTEN and TGF-β1 positivity (*r* = −0.306, *P* = 0.041). However, because of the small number of patients in the SSc and overlap syndrome groups, correlation analyses were not performed for these patients.

## Discussion

Sicca symptoms were more frequent in our current SSc patients (77%) than has been reported previously [18]. It should be noted, however, that all SSc patients examined in our present study had undergone MSGB as they had either sicca symptoms or autoantibody positivity suggesting an accompanying SS.

Our current findings suggest that the mTOR pathway might play an active role in the pathology of SS, SSc, and overlap syndrome. We did not find any statistically significant differences in the mTOR expression profile among our study groups indicating a common role of this pathway in these diseases. It may be appropriate, therefore, to use mTOR inhibitors more frequently in rheumatology practice.

We are not aware of any prior human study that has evaluated mTOR inhibitors in SS patients. Shah and colleagues have previously reported in a mouse model of SS that the mTOR inhibitor sirolimus may suppress the lymphocytic infiltration of lacrimal glands [11]. Our present observations may encourage other investigators to test mTOR inhibitors as potential new SS therapeutics.

Forestier et al. recently showed an altered B cell homeostasis in SSc patients compared with healthy controls [19]. This altered B cell homeostasis was found to be related to the mTOR pathway. However, the most important limitation of this study is the lack of a control group. Thus, further studies with healthy control groups may shed light on the pathogenesis of SSc and SS and may help us to understand the role of the mTOR pathway in the rheumatologic diseases.

We found a statistically significant difference between the acinar TGF-β1 expression levels in our study groups. As expected, strong TGF-β1 expression, a well-known fibrosis indicator, was found most frequently in the SSc patients. In addition, PTEN expression was similar in SS and SSc patients and this was significantly different from the overlap patients. These data suggest that epithelopathogenesis follows a different pathway in overlap syndrome than in SS or SSc. Although PTEN is known as an endogenous mTOR inhibitor, it has also previously been shown that increased mTOR activity is accompanied by increased PTEN levels and it was hypothesized that this might be due an autoimmune-related impairment in the PTEN pathway [7]. However, further studies are needed to investigate the effects of PTEN and PTEN-related molecules on autoimmune diseases.

Our correlation analysis in the SS group did not reveal any association between mTOR, PTEN, or TGF-β1 expression. This might indicate that PTEN and TGF-β1 operate independently of the mTOR pathway in SS pathogenesis. We also found a negative association between TGF-β1 and PTEN expression in our SS samples. Under normal physiological conditions in the cell, TGF-β1 may have both enhancing and reducing effects on PTEN. When the Ras/ERK pathway is activated, TGF-β suppresses PTEN by the SMAD4-independent signal pathway [7]. However, when the Ras/ERK pathway is inactivated, TGF-β upregulates the classic SMAD-dependent PTEN molecule [7]. The negative correlation between TGF-β1 and PTEN in SS suggests that the Ras/ERK pathway is active in this disease, but no precise information is yet available because other pathway members have yet to be identified. In addition, some notable limitations of our present study include the small number of SSc and overlap syndrome patients, the lack of sicca controls, and other reasons and technical difficulties with analyzing some of the biopsy materials.

## Conclusions

In conclusion, mTOR may be one of the common pathways leading to the pathology/inflammation observed in both SS and SSc and may provide a new alternative for the development of new treatments for both diseases. Additionally, higher PTEN and TGF-β1 expression, in particular a higher acinar TGF-β1 level, may be a distinctive feature of SSc.

## Key messages

- The mTOR pathway appears to be similarly active in minor salivary gland biopsies of SS and SSc patients.
- PTEN and TGF-β1 expression may be a distinctive feature of salivary gland pathology in SSc.

## Abbreviations

α-SMA: Alpha smooth muscle actin
ACR: American College of Rheumatology
AECG: American-European Consensus Group
Akt: Protein kinase B
AMP: Adenosine monophosphate
AMPK: Adenosine monophosphate-activated protein kinase
Bcl-2: B-cell lymphoma 2
BUT: Breakup time
CD4: Cluster of differentiation
eIF4E: Eukaryotic translation initiation factor 4E
ERK: Extracellular signal-regulated kinases
EULAR: European League Against Rheumatism
H&E: Hematoxylin and eosin
HLA: Human leukocyte antigen
IHC: Immunohistochemical
IL: Interleukin
JAK: Janus activated kinase
MAPK: Mitogen-activated protein kinase
mRNA: Messenger ribonucleic acid
MSGB: Minor salivary gland biopsy
mTOR: Mammalian target of rapamycin
mTORC1: Mammalian target of rapamycin complex 1
mTORC2: Mammalian target of rapamycin complex 2
NF-κB: Nuclear factor kappa B
PI3K: Phosphoinositol-3-kinase
PKB: Protein kinase B
PKC: Protein kinase C
PTEN: Phosphatase and tensin homolog
S6K1: S6 kinase 1
SD: Standard deviation
SMAD: Small mothers against decapentaplegic
SPSS: Statistical Package of Social Science
SS: Sjogren’s syndrome
SSc: Systemic sclerosis
STAT: Signal transducer and activator of transcription
TGF: Transforming growth factor
